# Editorial

**Published:** 1853-02

**Authors:** 


### THE MEDICAL EXAMINER.

PHILADELPHIA, FEBRUARY, 1853.

### TRIBUTE TO DR. BROWN-SÉQUARD

At a meeting of the medical gentlemen who have attended the lectures of Dr. E. Brown-Séquard, held Dec. 14th, 1852, Dr. Warren was called to the chair, and the following resolutions, offered by Dr. Henry J. Bigelow and seconded by Dr. Bowditch, were unanimously adopted, after amendments by Dr. Bartlett and Dr. Coale:

Resolved, That having attended a course of lectures by Dr. Brown-Séquard, illustrated by dissections and experiments upon subjects connected with physiology, and especially with that of the nervous system, we are desirous of expressing to Dr. Séquard the gratification which we have received from these lectures, which have been the result of extended original research, which have been eminently characterized by a spirit of rigid induction, and which have conveyed many practical hints upon pathological subjects. Therefore

Resolved, That we hereby offer our thanks to Dr. Séquard for the gratification and profit we have received from his very interesting lectures. That we commend these lectures to those members of our profession in other cities who may not have had the pleasure of hearing them; and that we indulge the hope that Dr. Séquard may find it convenient to continue his lectures here at some future time.

### CORRECTION.

The paper of Dr. Alison's, communicated by Dr. S. Lewis, in the January Number of this year, should have been accredited to the "Proceedings of the Royal Soc. of Edinburgh," instead of *Edinb. Phil. Trans.*

### NEW JERSEY MEDICAL INSTITUTE.

A new summer school has been established at Burlington, with which Dr. Joseph Parrish, Editor of the N. J. Medical Reporter, Dr. B. H. Rand, Dr. S. S. Brooks, Dr. J. B. Coleman and Dr. L. Read are associated. They offer many advantages to students during the summer season, and we doubt not will receive a full share of patronage. We wish them success.

CABINET OF MINERALS.

We call the attention of our readers to the following letter from Dr. Coxe. The collection is a very valuable one.

MY DEAR SIR,—May I request of you the favor to announce in your "Examiner" my intention of disposing of a very large and valuable Cabinet of Minerals, the collection of more than thirty years, and at an expense of several thousand dollars. The specimens are mostly of large size and beauty, and well adapted for a College or Public Institution. A large collection of models of the forms of crystals, constitute a part of the Cabinet; also, two large and fine specimens of hexagonal columns of Basalt, from the Giant's Causeway, in Ireland.

I am very truly your friend and obedient servant,

JOHN REDMAN COXE.

DR. SMITH.

*Jan.* 5, 1853.

---

At a stated meeting of the Northern Medical Association, held January 6, 1853, the following members were elected Officers for the present year:

*President*—Dr. Benjamin S. Janney.

*Vice President*—Dr. N. L. Hatfield.

*Counsellors*—Drs. Joseph R. Bryan, John F. Lamb, Wm. Mayburry, Isaac Remington and John Uhler, Jr.

*Secretary*—Dr. J. Henry Smaltz.

*Corresponding Secretary*—Dr. Isaac Remington.

*Reporting Secretaries*—Drs. John Rhein and C. P. Turner.

*Delegates to the American Medical Association*—Drs. L. Curtis, B. S. Janney, N. L. Hatfield, J. F. Lamb, R. J. Levis and Isaac Remington.

---

VIRGINIA MEDICAL AND SURGICAL JOURNAL is the title of a new aspirant for favor in Richmond, and edited by George A. Otis, M. D. and H. L. Thomas, M. D. The first number will be issued on April 1st, 1853, and the succeeding numbers will appear monthly.

The objects proposed in its publication are the diffusion of *Medical Knowledge* and *Intelligence;* the advancement of *Science;* and the support of the dignity and interests of the Medical Profession.

Each number will consist of eighty large octavo pages, containing Original Papers, Memoirs, and Reports of cases; an account of the transactions of Medical Societies in Virginia; a Summary of Improvements and Discoveries in Medical Science, at home and abroad; Reviews and

Bibliographical Notices; and a portion of a Standard Work in some important Department in Medicine. Price, $5 a year.

We welcome our new friends into the editorial fraternity, and assure them of our hearty wishes for their success. There is material and talent enough, and to spare, in the Old Dominion to support and encourage another good Journal.

